# Supplementary material for: Problems of magnetic resonance diagnosis for gastric-type mucin-positive cervical lesions of the uterus and its solutions using artificial intelligence
Source: PLoS One. 2024 Dec 30;19(12):e0315862. doi: 10.1371/journal.pone.0315862 (PMC11684648; doi:10.1371/journal.pone.0315862)
Supplement: Attachment — Submitted filename: Comments to the Author.docx [file pone.0315862.s001.docx]

Comments to the Authors

This study used MRI images to differentiate gastric mucin-positive (GMPL) from gastric mucin-negative (GMNL) to validate diagnostic performance and concordance rates by three sets of physicians and a CNN.

The methodology is valid, but there are significant parts that would benefit from substantial revision.

Overall

・In academic or formal writing, especially in scientific papers, "physician" is often preferred over "doctor" when referring to medical doctors.

・The values for metrics such as AUC and ICC are inconsistent; it would be better to standardize them to three decimal places.

・It is confusing because 'validation' and 'test' are used interchangeably; it would be better to use 'test' consistently.

・Independent evaluation of six readers and additional external testing is recommended.

Abstract

・The purpose and conclusion are not aligned. The solution is not clear.

・(axial T2-weighted image [T2WI], axial T1-weighted image [T1WI], and sagittal T2WI)→Please use the same order of notation as in the text.

・I think it is common to express diagnostic ability using AUC.

・Since the same model and the same test data are used, it is natural that the CNN has high reproducibility.

Introduction

・P.5 Line 56, Some reports have described adenocarcinomas in association with LEGH [5–8], does it mean adenocarcinoma other than GAS?

・P.6 Line 1-71, It is redundant and should be concise.

・P.7 Line 87-90, In addition, since artificial intelligence always makes the same diagnosis once it learns, it can be a promising solution when the reproducibility of doctors’ diagnoses is low.→As for CNN, doesn't it make sense to evaluate reproducibility on different test sets?

M&M

MR images

・As Figure 1, patient selection, diagnostic rationale, and assignment to training and testing should be presented in a clear manner.

・P.8 Line 104, ...other than LEGH, LEGH, LEGH with→Isn't there a need for a LEGH in the middle?

・P.8 Line 110, First appearance of BCL is to be spelled out.

・Lesion’s classification should also be tabulated.

・P.10 Line 131, minimum imaging conditions should be described, such as slice thickness, whether 2D or 3D, and the cross-section of imaging (e.g., orthogonal to the cervix).

Diagnostic accuracy and reproducibility by the readers

・Why did you choose to use consensus by pairs? It would be more objective to do each independently and give data for the six people separately.

・What is the definition of diagnostic confidence?

・Is it correct in understanding that they evaluated the same test data with trimming that you put into the CNN?

・Did they evaluate the images without checking for image features that distinguish GMPL from GMNL?

Diagnostic accuracy evaluation by pCNNs

・P.11 Line 139, please list the years of experience of the readers.

・P.11 Line 155, Xception requires a citation

・P.12, Line167, if you are using 3 combined images, I don't think rotation makes sense

Fig1.

Figure 1 is not well explained, but did you use the 3 images as a combined image and each sequence was always trained and tested in the same position and in different colors?

Fig 2.

①②③④ means layers?

I'm not sure, so why don't you change it so we can see which is the same training set, e.g. T①, and leave the current ① out?

Statistical analysis

・P.14 Line 202, Since kappa value comes out of nowhere, it should be explained in the statistics section if you want to bring it out.

Results

・Please describe the characteristics of the patient group (age, etc.) in the first section.

・First, second, ... in Tables 1, 2 and 3 should be unified.

Fig 3.

(A) Comparison of receiver operating characteristic (ROC) curves at the time of the second independent procedure was entered in Xception and at the time of the first interpretation experiment of pair A with the lowest area under the curve (AUC) among the readers.→What made this one so big out of so many?

The former AUC was 0.8535, which was higher than the latter AUC (0.7197).→Please specify what former and latter refer to.

Comparison of ROC curves at the time of the first independent procedure of Xception and　at the time of the second interpretation experiment of pair A with the highest AUC value. →What made this one so big out of so many?

The former AUC was 0.8409, which was lower than the latter AUC (0.8144). →Please specify what former and latter refer to.

Discussion

・P.22, Line 8, How are the diagnostic methods different?

・Please explain why one AUC for an experienced radiologist is lower than for a non-experienced radiologist.

・The value of ICC among experienced radiologists is of interest and should have been evaluated independently for each of the six readers.

P.25, Line 7-8. If the same training data are used, a highly reproducible diagnosis can be established regardless of facilities. →Although a variety of models are included in the training, reproducibility with models not included in the training has not been tested, so this mention cannot be made.

P.25, Line 8-9. Current problems in the MR diagnosis of GMPLs and GMNLs may be solved by pCNNs.→Since no significant difference was found, this reference is also questionable.
