# Supplementary material for: Problems of magnetic resonance diagnosis for gastric-type mucin-positive cervical lesions of the uterus and its solutions using artificial intelligence
Source: PLoS One. 2024 Dec 30;19(12):e0315862. doi: 10.1371/journal.pone.0315862 (PMC11684648; doi:10.1371/journal.pone.0315862)
Supplement: Attachment — Submitted filename: point_by_point_response ver.3-2.docx [file pone.0315862.s002.docx]

**Response to the reviewers’ comments**

We deeply appreciate the reviewers’ educational suggestions. According to the suggestions, we have revised our manuscript as follows. The revised sentences are displayed as red letters in the revised manuscript.

**Reviewer #1:** Comments to the Authors

This study used MRI images to differentiate gastric mucin-positive (GMPL) from gastric mucin-negative (GMNL) to validate diagnostic performance and concordance rates by three sets of physicians and a CNN.

The methodology is valid, but there are significant parts that would benefit from substantial revision.

Overall

・In academic or formal writing, especially in scientific papers, "physician" is often preferred over "doctor" when referring to medical doctors.

**Response:** Thank you for pointing this out. We have revised the term “doctor(s)” to “physician(s)” as per your suggestion. (P6L73, P7L83, L86, Table 3 L250, P24L306, L307, P26L337, L338, L340, P27L362 in the revised manuscript)

“vary from doctor to doctor” was revised to “vary among physicians” (P25L319 in the revised manuscript)

・The values for metrics such as AUC and ICC are inconsistent; it would be better to standardize them to three decimal places.

**Response:** We completely agree with your comment. We have unified the AUC and ICC values to three decimal places. (P4L42, P17L239, L240, L242, Table 2, Table 3, P20L259, L264, Table 4, P21L277, P22L279, L282, L284 in the revised manuscript)

・It is confusing because 'validation' and 'test' are used interchangeably; it would be better to use 'test' consistently.

**Response:** To avoid confusion among readers, the term “validation” is used in both the text and figure legend.

- “validation test” to “validation” (P12L166, P13L171 in the revised manuscript)
- “test” to “validation” (P15L203, L205, P16L218 in the revised manuscript)

・Independent evaluation of six readers and additional external testing is recommended.

**Response:** As per your suggestion, we conducted additional experiments. The results demonstrated a diagnostic accuracy of up to 80% and an AUC of up to 0.859. Diagnostic accuracy of the readers was slightly lower than that of CNN, and AUC of the readers was slightly higher than that of CNN; however, the 95% confidence intervals overlapped and were not significantly different. The following are the results of additional experiments.

| Reader | Diagnostic accuracy | AUC (95% CI) |
| --- | --- | --- |
| A | 0.75 | 0.859 (0.746–0.972) |
| B | 0.7 | 0.797 (0.658–0.936) |
| C | 0.675 | 0.705 (0.540–0.869) |
| D | 0.8 | 0.816 (0.678–0.953) |
| E | 0.775 | 0.793 (0.646–0.940) |
| F | 0.7 | 0.729 (0.566–0.891) |

Overall, both the diagnostic accuracy and AUC slightly improved when the reading experiment was conducted individually compared to when it was performed in pairs. However, the diagnostic accuracy of the individual is slightly lower than that of the CNN, and the individual AUC is not statistically significantly different from the CNN AUC. The same is the case for the pair.

***Abstract***

・The purpose and conclusion are not aligned. The solution is not clear.

**Response:** Based on your valuable comment, we have aligned our objectives and conclusions. The first sentence of the conclusion has been changed as follows: Variation in the inter-reader or intra-reader accuracy in MRI diagnosis limits differentiation between GMPL and GMNL. (P4L43-44 in the revised manuscript)

・(axial T2-weighted image [T2WI], axial T1-weighted image [T1WI], and sagittal T2WI)→Please use the same order of notation as in the text.

**Response:** As per your comment, we have ensured the dame order of notation as in the text. (P3L31-32 in the revised manuscript)

・I think it is common to express diagnostic ability using AUC.

**Response:** We completely agree with your comment. Therefore, the first sentence of the conclusion has been changed as follows: Variation in the inter-reader or intra-reader accuracy in MRI diagnosis limits differentiation between GMPL and GMNL. (P4L43-44 in the revised manuscript)

・Since the same model and the same test data are used, it is natural that the CNN has high reproducibility.

**Response:** In CNN, the same model and the same test data are not necessarily reproducible. This is because even if the CNN uses the same model, different training methods may lead to different test results. Many papers do not confirm the reproducibility of the results by CNN. Thus, we have demonstrated that CNN is highly reproducible, even if it trains on the same data in different ways.

***Introduction***

・P.5 Line 56, Some reports have described adenocarcinomas in association with LEGH [5–8], does it mean adenocarcinoma other than GAS?

**Response:** We greatly appreciate your point of view. We have deleted that sentence. (P5L53 in the revised manuscript)

・P.6 Line 1-71, It is redundant and should be concise.

**Response:** Thank you for your suggestion.

“Various benign lesions with cystic components occur in the uterine cervix. Common benign lesions include Nabothian cysts, tunnel clusters, lobular endocervical glandular hyperplasia (LEGH), endometriosis, and cervical polyps [1]. LEGH is a hyperplastic benign lesion first proposed by Nucci et al. [2], but it may be a precursor lesion for gastric-type mucinous carcinomas (GAS) with a very poor prognosis [3,4]. Some reports have described adenocarcinomas in association with LEGH [5–8]. The most recent study demonstrated that malignant change of LEGH occurs at a frequency of 1.4% [9], but frequent and long-term follow-up or surgical treatment is selected once LEGH is diagnosed. In contrast, other benign cystic lesions of the uterine cervix do not require follow-up as frequently as LEGH. Therefore, clinically, LEGH and other benign cystic lesions must be distinguished.

LEGH has been reported to exhibit magnetic resonance imaging (MRI) findings called ‘cosmos pattern’, while Nabothian cysts show coarse cysts pattern [10]. However, some Nabothian cysts exhibit MR findings similar to those of LEGH [11]. The decisive difference from other benign lesions is that LEGH and GAS exhibit a distinctive pyloric gland metaplasia and secrete gastric-type mucin, which has O-linked oligosaccharides with a terminal α1,4-linked N-acetylglucosamine (αGlcNAc) residue [12]. Because gastric-type mucin is a neutral mucin, LEGH and GAS exhibit a ‘two-colour pattern’ on Pap smears [13]. Further, αGlcNAc expression exhibits positive immunohistochemical findings for monoclonal antibody HIK1083. αGlcNAc has been detected in cervical mucus by latex agglutination assay using monoclonal antibody HIK1083 [14].” was revised to

“Common benign lesions in the uterine cervix include Nabothian cysts, tunnel clusters, lobular endocervical glandular hyperplasia (LEGH), endometriosis, and cervical polyps [1]. LEGH is a benign lesion first proposed by Nucci et al. [2], but it may be a precursor lesion for gastric-type mucinous carcinomas (GAS) [3–8]. Therefore, frequent and long-term follow-up or surgical treatment is selected once LEGH is diagnosed. In contrast, other benign cystic lesions of the uterine cervix do not require follow-up as frequently as LEGH. Therefore, clinically, LEGH and other benign cystic lesions must be distinguished.

LEGH shows magnetic resonance imaging (MRI) findings called ‘cosmos pattern’, while Nabothian cysts show coarse cysts pattern [9]. However, some Nabothian cysts exhibit MR findings similar to those of LEGH [10]. The decisive difference from other benign lesions is that LEGH and GAS secrete gastric-type mucin, which has O-linked oligosaccharides with a terminal α1,4-linked N-acetylglucosamine (αGlcNAc) residue [11]. Because gastric-type mucin is a neutral mucin, LEGH and GAS exhibit a ‘two-color pattern’ on Pap smears [12]. Further, αGlcNAc has been detected in cervical mucus by latex agglutination assay using monoclonal antibody HIK1083 [13].” (P5L50-P6L65 in the revised manuscript)

・P.7 Line 87-90, In addition, since artificial intelligence always makes the same diagnosis once it learns, it can be a promising solution when the reproducibility of doctors’ diagnoses is low.→As for CNN, doesn't it make sense to evaluate reproducibility on different test sets?

**Response:** Thank you for your comments. Physicians used the same validation data to determine reproducibility. The CNN would have to be evaluated under the same conditions to be comparable. In addition, because GMPLs are relatively rare diseases, it has been impossible to provide separate validation datasets.

***M&M***

MR images

・As Figure 1, patient selection, diagnostic rationale, and assignment to training and testing should be presented in a clear manner.

**Response:** We completely agree with your comment. We have added a new Figure 1 and its caption to make patient selection, diagnostic rationale, and assignment to training and testing easier to understand. Therefore, Figure numbers were rearranged. “The process of patient selection, and grouping is summarized in Fig 1.” was added. (P7L95-96 in the revised manuscript)

・P.8 Line 104, ...other than LEGH, LEGH, LEGH with→Isn't there a need for a LEGH in the middle?

**Response:** Yes, there is. For clarity, “(BCL)” was added after “other than LEGH”. (P8L101 in the revised manuscript)

・P.8 Line 110, First appearance of BCL is to be spelled out.

**Response:** As noted above, “BCL” is already exists at P8L101.

・Lesion’s classification should also be tabulated.

**Response:** As per your suggestion, we have summarized the pathological features of the lesion in new Table 1. Therefore, we rearranged Table numbers.

“The classification of histopathologic lesions is summarized in Table 1.” was added at P9L113-114 in the revised manuscript.

・P.10 Line 131, minimum imaging conditions should be described, such as slice thickness, whether 2D or 3D, and the cross-section of imaging (e.g., orthogonal to the cervix).

**Response:** As you have accurately pointed out, we have added basic information about the image as follows: All images have a slice thickness of 2 to 7.5 mm and are captured as two-dimensional images. (P12L161-162 in the revised manuscript)

***Diagnostic accuracy and reproducibility by the readers***

・Why did you choose to use consensus by pairs? It would be more objective to do each independently and give data for the six people separately.

**Response:** The CNN performs validation using four differently trained folds. Therefore, we considered that humans should also be examined by more than one group to be considered as equals, so we examined them in pairs. Additional independent studies were conducted for each of the six individuals.

・What is the definition of diagnostic confidence?

**Response:** The confidence level of the diagnosis was stated as a percentage based on each physician's experience. We have appended this statement as follows: The confidence level of the diagnosis was stated as a percentage based on each physician’s experience. (P13L173-174 in the revised manuscript)

・Is it correct in understanding that they evaluated the same test data with trimming that you put into the CNN?

**Response:** Yes, it is.

・Did they evaluate the images without checking for image features that distinguish GMPL from GMNL?

**Response:** Yes, they did. They did not have information of useful image features that distinguished GMPL from GMLN when they evaluated the images.

***Diagnostic accuracy evaluation by pCNNs***

・P.11 Line 139, please list the years of experience of the readers.

**Response:** As per your comment, we have added the years of experience of each reader. (P12L169-P13L170 in the revised manuscript)

・P.11 Line 155, Xception requires a citation

**Response:** As per your comment, we have added the paper of Xception to the references (ref# 15).

・P.12, Line167, if you are using 3 combined images, I don't think rotation makes sense

**Response:** When the three images are superimposed, the angle of rotation is limited to 10° at random. Rotation is not unnatural because the cervix is tilted differently in each person. This method is common and used previous reports (La Grace Saint-Esteven A, et al. Comput Biol Med. 2022 142: 105215).

***Fig1.***

Figure 1 is not well explained, but did you use the 3 images as a combined image and each sequence was always trained and tested in the same position and in different colors?

**Response:** CNN recognizes three images by inputting them into three color channels. We input a T2-weighted sagittal section in the red channel, a T2-weighted transverse section in the green channel, and a T1-weighted transverse section in the blue channel. We ensured that the images were always entered in the same way during training and during validation.

***Fig 2.***
①②③④ means layers?

I'm not sure, so why don't you change it so we can see which is the same training set, e.g. T①, and leave the current ① out?

**Response:** ①②③④　are the training data randomly divided into four parts; of the four parts, three colored data are used for training and the uncolored data are used for provisional validation. CNN training uses three of these four divisions of data to train four different types of training.

***Statistical analysis***

・P.14 Line 202, Since kappa value comes out of nowhere, it should be explained in the statistics section if you want to bring it out.

**Response:** Thank you for pointing this out. This is an error. We have corrected “kappa value” to “ICC (1) or (2)”. (P16L225 in the revised manuscript)

***Results***

・Please describe the characteristics of the patient group (age, etc.) in the first section.

**Response:** We are unable to describe the characteristics of the patients in the Results section because they are not part of the survey. The age of the patients is described in the “Patient population” section as follows: Patients ranged in age from 26 to 82 years, with an average age of 48.7 years. (P7L94-95 in the revised manuscript)

・First, second, ... in Tables 1, 2 and 3 should be unified.

**Response:** As per your comments, we have changed Table 3 to unify them.

***Fig 3.***

- Comparison of receiver operating characteristic (ROC) curves at the time of the second independent procedure was entered in Xception and at the time of the first interpretation experiment of pair A with the lowest area under the curve (AUC) among the readers.→What made this one so big out of so many?

**Response:** We selected and enlarged this graph because we compared the highest AUC in Xception to the lowest AUC for the physician pair.

- The former AUC was 0.8535, which was higher than the latter AUC (0.7197).→Please specify what former and latter refer to.

**Response:** Thank you for pointing this out. It was difficult to understand, so we have corrected the relevant part as follows: The AUC at the time of second independent procedure of Xception was 0.854, which was higher than the AUC at the time of the first interpretation experiment of pair A (0.720) (P21L277-P22L279 in the revised manuscript)

- Comparison of ROC curves at the time of the first independent procedure of Xception and at the time of the second interpretation experiment of pair A with the highest AUC value. →What made this one so big out of so many?

**Response:** We selected and enlarged this graph because we compared the lowest AUC in Xception to the highest AUC for the physician pair.

- The former AUC was 0.8409, which was lower than the latter AUC (0.8144). →Please specify what former and latter refer to.

**Response:** Thank you for pointing this out. To make the sentence easier to understand, we have corrected the relevant part as follows: The AUC at the time of the first independent procedure of Xception was 0.841, which was lower than the AUC at the time of the second interpretation experiment of pair A (0.814). (P22L282-284 in the revised manuscript)

***Discussion***

・P.22, Line 8, How are the diagnostic methods different?

**Response:** We have added the following sentences. “Although radiologists are experts in diagnostic imaging, gynecologists make a comprehensive diagnosis based on the clinical symptoms and other clinical information. Since clinical information could not be gathered in this experiment, the accuracy of the gynecologists was considered lower than that of the radiologists.” (P24L310-314 in the revised manuscript)

・Please explain why one AUC for an experienced radiologist is lower than for a non-experienced radiologist.

**Response:** We are unable to explain this as it is the result of a serious experiment. Pair A had a lower AUC than Pair B the first time but a higher AUC the second time. The AUCs were not significantly different, overlapping 95% confidence intervals for all readers.

・The value of ICC among experienced radiologists is of interest and should have been evaluated independently for each of the six readers.

**Response:** Based on the results of additional experiments, the value of ICC among experienced radiologists is 0.679. ICC results are shown below. The ICC (2, 1) for each of the six combinations indicated a maximum of 0.679 and a minimum of 0.359, which did not vary considerably when evaluated in pairs. Thus, adding the results of the six independently enforced results does not change the conclusion. We decided not to add the description of this additional study because it would have resulted in considerable revision of the paper in the methods and results sections.

ICC (2,1)

| Reader | B | C | D | E | F |
| --- | --- | --- | --- | --- | --- |
| A | 0.679 | 0.458 | 0.454 | 0.554 | 0.509 |
| B |  | 0.451 | 0.310 | 0.421 | 0.615 |
| C |  |  | 0.562 | 0.359 | 0.520 |
| D |  |  |  | 0.557 | 0.605 |
| E |  |  |  |  | 0.517 |

- P.25, Line 7-8. If the same training data are used, a highly reproducible diagnosis can be established regardless of facilities. →Although a variety of models are included in the training, reproducibility with models not included in the training has not been tested, so this mention cannot be made.

**Response:** Since the validation data were not used for training, we can refer to the reproducibility of the CNN.

- P.25, Line 8-9. Current problems in the MR diagnosis of GMPLs and GMNLs may be solved by pCNNs.→Since no significant difference was found, this reference is also questionable.

**Response:** Diagnostic performance was the same; however, CNN outperformed physicians in reproducibility. The problem with the current GMPL and GMNL diagnoses is that the diagnosis is likely to vary among physicians, as demonstrated by our study. CNNs with high reproducibility can ensure uniformity of diagnosis if physician diagnosis and diagnostic performance are the same.

**Reviewer #2**

The focus of your research is excellent. As noted, LEGH and other benign cystic lesions must be distinguished.

Since this is 20 years of data, there are considerable differences in reading due to the imaging accuracy of MRI equipment and other factors.

In addition, it would be helpful to consider at what point the reading differed for cases in which there was a discrepancy in diagnosis in both the Human and CNN groups, to improve the agreement rate.

Although it is unclear how widespread the use of convolutional neural networks will become in the future, the significant difference in the positive diagnosis rate compared to human reading showed that it is a useful tool.

CNN is still not very popular in Japan, but if it becomes popular, it will be applied to preoperative diagnosis of gynecological tumors as well as LEGH, and this is a very interesting paper.

***Materials and Methods:***

{clinical suspicion of LEGH or GAS (based on ultrasonographic findings and symptoms)}

Please describe in detail what the ultrasound findings and symptoms are specifically.

**Response:** As per your comments, we have added “such as multiple cysts of the cervix” as ultrasonographic findings and “such as vaginal watery discharge” as symptoms. (P7L92-93 in the revised manuscript)

***MR images:***

{The types and magnetic field strength of the MR unit used for imaging and the parameters of each sequence varied because the study was retrospective and MR images were acquired over a long period.}

Since the observation period is as long as 20 years and the MRI equipment must have changed many times during that time, I think that the bias by equipment is quite large.

At the very least, please consider how many times the MRI equipment has changed, as well as the details of that equipment and the statistics by equipment.

**Response:** Thank you very much for pointing this out. The number of MRI machines used and the number of patients imaged with each machine have been added to the “MR images” section.

“All the patients underwent MRI using 1.5 or 3.0 tesla scanners. A total of 134 patients underwent MRI by Siemens scanners (Siemens Healthcare Diagnostics, Erlangen, Germany) – 28 patients by 3T Trio, 27 patients by 3T Prisma, 22 patients by 3T Vida, 2 patients by 3T Skyra, 39 patients by 1.5T Avanto, 11 patients by 1.5T Symphony, three patients by 1.5T Aera, and two patients by 1.5T Essenza. A total of 29 patients underwent MRI by GE scanners (GE HealthCare, Chicago, Illinois, USA) – two patients by 3T DISCOVERY MR 750w, one patient by 3T SIGNA Pioneer, one patient by 1.5T OPTIMA MR360w, 15 patients by 1.5T OPTIMA MR450w, 10 patients by 1.5T SIGNA HDxt, and one patient by 1.5T SIGNA Excite HD. The remaining nine patients were imaged with two types of 3T MRI scanners (Philips Electronics N.V., Amsterdam, Holland; Canon Medical Systems Corp., Tochigi, Japan) and three types of 1.5T MRI scanners (Philips Electronics N.V., Amsterdam, Holland; Canon Medical Systems Corp., Tochigi, Japan).” (P11L149-P12L161 in the revised manuscript)

***Diagnostic accuracy and reproducibility by the readers：***

{divided into the training (132 patients) and validation (40 patients) test groups}

Please describe the reasons for any differences in the number of patients between groups.

**Response:** A new Figure 1 and the legends have been added (P9L121-P10L135 in the revised manuscript), and the reason for the difference in the number of people in the training and validation groups was described in the legend of the Figure as follows. “Training data are more numerous than validation data because more data are needed to train the CNN.” (P9L134-135 in the revised manuscript) Following Figure numbers were rearranged.

***Discussion:***

{The second limitation is that it was not possible to analyse what MR features Xception recognised and diagnosed.}

In both Human and CNN groups, please describe the basis for reading LEGH from the MRI images.

**Response:** Physicians are likely to base their decisions on whether the lesions are in the cosmos pattern or not. However, we do not know what CNN learned from the training data to arrive at its answer, because the CNN averaged the results produced by the four folds to arrive at the final result. The following is described in the Discussion section. “Physicians are likely to base their decisions on whether the lesions are in the cosmos pattern or not. However, we averaged the diagnostic probabilities of four-fold cross-validation to avoid overfitting, preventing us from analysing where Xception focused on the MR image.” (P27L351-354 in the revised manuscript)

***Conclusion:***

{In conclusion, our study revealed MR diagnostic heterogeneity among the readers in distinguishing between GMPLs and GMNLs.}

I think it is impossible to discuss the concordance rate without considering at what point the differences in reading were observed in the cases with discrepancies in diagnosis in both the Human and CNN groups.

**Response:** In the case of humans, the experiment is performed in multiple pairs, and it is administered twice. The low concordance rate of the answers also made it difficult to identify cases in which the human pairs were commonly wrong and the CNNs were correct. We also consider it important to verify the difference between human and CNN readings, but at this point we have not decided on a method of verification. Therefore, it is not possible to verify the differences between humans and CNNs at this time. We consider this an issue for future consideration.

In addition, we have revised as follows;

- “Gynaecology” or “gynaecologist” were revised to “gynecology” or “gynecologist”.
- “Colour” to “color”
- “analyse” to “analyze”
- Because “Cica HIK gastric-type mucin” is the trade name, we change it to “HIK test”. (P8L97, P26L345, L346, L348 in the revised manuscript)
- “The Cica HIK gastric mucin test used as the final result has a very high sensitivity and specificity” was revised to “The HIK1083-latex agglutination assay (HIK test), used as the final result, has a very high sensitivity and specificity for detecting gastric-type mucin” (P26L342-343 in the revised manuscript)
- “Data Availability Statement” was added at P28L378-380.
