# Supplementary material for: Problems of magnetic resonance diagnosis for gastric-type mucin-positive cervical lesions of the uterus and its solutions using artificial intelligence
Source: PLoS One. 2024 Dec 30;19(12):e0315862. doi: 10.1371/journal.pone.0315862 (PMC11684648; doi:10.1371/journal.pone.0315862)
Supplement: Attachment — Submitted filename: point by point response R2.3-3.docx [file pone.0315862.s003.docx]

**Response to the reviewers’ comments**

We deeply appreciate the reviewers’ educational suggestions. According to the suggestions, we have revised our manuscript as follows. The revised sentences are displayed as red letters in the revised manuscript.

**Reviewer #1:**

Thank you for the revision according to the comments. I understand that there is no significant difference in the results whether in pairs or individually.
Please consider the following minor revisions.

Abstract
P.3, line 36, (sagittal T2WI and axial T2WI/T1WI) may be better.

**Response:** Thank you for pointing this out. We have corrected it, as you suggested.

Overall: Since "magnetic resonance imaging (MRI) findings" is mentioned in Introduction p.5, line 58, I think it is better to use "MRI findings", "MRI diagnosis", and "MR unit" instead of "MR findings", "MR diagnosis", and "MRI unit" thereafter. However, "MR images" can remain as is.

**Response:** Thank you for pointing this out. We have corrected it, as you suggested.

Table 1 should include other BCLs with histologically unconfirmed diagnosis, as well as information on the number of patients and their ages. The title should be changed accordingly.

**Response:** Thank you for your feedback. We have revised Table 1 and its title, as you suggested.

MR images
P.12, L.168, For the CNN, it is assumed that one image per patient was used, so it would be better to add "per patient" as in: "One image showing the maximum cross-section of the lesion was selected from each of these three sequences per patient, and the three images were used for analysis."

**Response:** Thank you for pointing this out. We have added “per patient”, as you suggested.

Diagnostic accuracy and reproducibility by the readers
P.12, L168, GMNL. (Fig 1)→Move the period to the end.

**Response:** We apologize for our careless mistake. We have corrected it, as you suggested.

Diagnostic accuracy evaluation by pCNNs
P.14, L189, the validation group. (Fig 1)→Move the period to the end.

**Response:** We apologize for our careless mistake. We have corrected it, as you suggested.

P.14, L200, I think "(Fig 2)" can be omitted from "Using the training data, all three images were input into Xception."

**Response:** Thank you for pointing this out. We have corrected it, as you suggested.

Fig2. "Input" is used for converting Fig into color, and it may cause confusion with "Input" for CNN in the main text. Therefore, I think it would be better to change the text in the figure to something like "Conversion."

**Response:** Thank you for your suggestion. As you said, Fig 2 shows that a black and white images are inputted and consequently converted into color images. We have revised Fig 2, as you suggested. In addition, we have revised the Figure Legends of Fig 2 as follows.

“A sagittal T2-weighted image was input into the red channel, an axial T2-weighted image was input into the green channel, and a T1-weighted image was input into the blue channel. These converted images were processed to include only the lesion and cervical stroma of the uterus.” (P16 L214-217 in the revised manuscript)

Fig3. The "test" in the figure and "the validation data" in the figure description may cause confusion with cross-validation, so I think it would be better to unify them as "independent validation".
The first letters in the abbreviation explanations of Fig 3 should be consistently either capitalized or in lowercase.

**Response:** According to your suggestion, we have revised Fig 3, and its Figure legends.

Diagnostic accuracy evaluation by pCNNs
P.15, L203-205
“Finally, images of the validation data were input into the model trained in each fold, and the true diagnostic accuracy and AUC for the validation data were determined by averaging the diagnostic probabilities by the four models (Fig 3).”
I think using "independent validation" would make the above sentences clearer.

**Response:** Thank you for your feedback. Your point is certainly valid. According to your suggestion, we have added “independent” at P15L207 and L208 in the revised manuscript.

Table 3. The notation in the "Second" column of Pair B is incorrect.

**Response:** We apologize for our careless mistake. We have corrected it.

In the Figure legends of Fig 4, please standardize the decimal places for the p-values to three digits, same as the others. “However, there was no statistically significant difference between the two (p =0.0939).” “However, there was no statistically significant difference between the two (p =0.6813).”

**Response:** According to your suggestion, we have revised 0.0939 to 0.094, and 0.6813 to 0.681. (L285 and L290 in the revised manuscript)

Table 4. It would be better to include the meaning of "Tentative diagnostic accuracy" in the main text.

**Response:** Thanks for your suggestion. We have corrected the sentence on P14L204-206 in the revised manuscript as follows: The accuracy of each verification by the four-fold cross-validation was calculated and averaged to the tentative diagnostic accuracy, which indicates degree of training.

Please change the lower limit value of the Tentative diagnostic accuracy for "second" to three decimal places.

**Response:** Thanks for your point of view. We have corrected the relevant area.

Discussion
P.25. L.327-331. Since "readers" appears consecutively, I think the second "readers" can be omitted.

**Response:** According to your suggestion, “Additionally, the precision, specificity, and F-measure were all higher than those of the readers” was revised to “Additionally, the precision, specificity, and F-measure were all higher.” (P26L333 in the revised manuscript)

**Reviewer #2:**

Since CNN is almost as accurate as the reader, it has not shown usefulness in reading at this time. It has little IMPACT as a new FINDINGS. In addition, CNN may have the potential to improve diagnostic reproducibility, but in actual clinical practice, when a treatment plan is decided based on MRI readings, “diagnostic reproducibility” is not as useful as “diagnostic accuracy. I don't feel it is useful.

**Response:** We understand your point of view. However, this study aims to explore the issues in the diagnosis of LEGH, demonstrating that current human MRI diagnoses of LEGH are inconsistent and that the interpretation results can vary significantly depending on the reader. Diagnosis by the CNN was only used as a comparison to verify the variability of human diagnosis, and it is not the purpose of this study to verify the superiority of CNN. We agree that the diagnostic accuracy is important. However, we believe that the reproducibility is equally important, as low reproducibility includes the possibility that accuracy may be reduced if a different doctor diagnoses the patient.

What would you estimate the number of LEGH clinical experiences of the readers to be for a CNN that has trained 132 LEGH cases? For example, I think it is much more experienced than the group of young radiologists. What do you consider the difference?

**Response:** There is a misunderstanding in your interpretation. A CNN cannot provide an adequate diagnosis unless it is trained on around 100 cases. On the other hand, the 'young radiologist' in this study, though less experienced with LEGH, is a specialist in diagnostic imaging. The fact that the pairs of young radiologists achieved the highest diagnostic accuracy cannot be ignored. Since there was no significant difference in accuracy between the CNN and the radiologists, it can be concluded that even though the young radiologists did not have prior training, they were not inferior to the CNN.

As Reviewer 1 also pointed out, I don't understand the significance of examining the pairs. I think a comparison between individuals would be better; you have also disclosed the results between individuals in Response, and this should be noted and discussed.

**Response:** In response to Reviewer 1’s comments, we disclosed the individual physicians’ reading results, but there was no difference compared to when the readings were done in pairs. Therefore, we did not include it in the manuscript. Additionally, the CNN provides final results by averaging the outcomes of the four folds. Since CNN is using an average of 4 folds to answer the question, we thought the physicians should also be in the group.

The group of readers did not study the LEGH cases in advance and made decisions based on their limited clinical experience, which may be a disadvantage compared to the CNN, which is supposed to be studied in advance.

**Response:** We agree with your comment. If the readers had been trained beforehand, they might have had a better accuracy. However, as mentioned above, diagnosis by the CNN was only used as a comparison to verify the variability of human diagnosis, and it is not the purpose of this study to verify the superiority of CNN.

Isn't it a leap to conclude that a CNN's finding of a significant difference in diagnostic reproducibility can provide the same level of diagnosis everywhere as a reading physician, when no significant difference in diagnostic accuracy has been found?

**Response:** What we would like to say is that when a certain finding was found, humans may vary in their diagnosis, but CNN do not, making them useful as a criterion for diagnosis. It goes without saying that a high accuracy and reproducibility are ideal.

Without analysis of what MRI features CNNs recognized and diagnosed, I don't think this will lead to the widespread use and improvement of CNNs in the future.

**Response:** Thank you for your important comment. We agree your comment. We also believe it is important to clarify what features of MRI images the CNN captures and understands for its development. However, the specific details of how the CNN analyzes images are not clearly established, so it is even unclear if it operates in the same way as human interpretation. Additionally, in this study, the CNN uses the average results of the four folds as the final outcome, and due to the low agreement rate among physicians across the three groups, the analysis and comparative evaluation are very complex and challenging. We think that exploring how the CNN recognizes MRI images needs to be addressed through a different approach and should be a focus for future studies.
